# Proton Pump Inhibitors and Cognitive Health: Review on Unraveling the Dementia Connection and Co-morbid Risks

**DOI:** 10.2174/0115672050289946240223050737

**Published:** 2024-04-09

**Authors:** Zuber Khan, Sidharth Mehan, Mohd. Anas Saifi, Ghanshyam Das Gupta, Acharan S. Narula, Reni Kalfin

**Affiliations:** 1 Division of Neuroscience, Department of Pharmacology, ISF College of Pharmacy, Moga, Punjab, India (Affiliated to IK Gujral Punjab Technical University), Jalandhar, Punjab, 144603, India;; 2 Department of Medical Elementology and Toxicology, School of Chemical and Life Sciences, Jamia Hamdard, New Delhi-110062, India;; 3 Department of Pharmaceutics, ISF College of Pharmacy, Moga, Punjab, India (Affiliated to IK Gujral Punjab Technical University), Jalandhar, Punjab, 144603, India;; 4 Narula Research, LLC, 107 Boulder Bluff, Chapel Hill, NC 27516, USA;; 5 Institute of Neurobiology, Bulgarian Academy of Sciences, Acad. G. Bonchev St., Block 23, Sofia 1113, Bulgaria;; 6 Department of Healthcare, South-West University “NeofitRilski”, Ivan Mihailov St. 66, Blagoevgrad 2700, Bulgaria

**Keywords:** Proton-pump inhibitor, dementia, amyloid-beta, neurodegeneration, adverse effects, amyloid-beta (Aβ), peptides

## Abstract

Dementia, an international health issue distinguished by the impairment of daily functioning due to cognitive decline, currently affects more than 55 million people worldwide, with the majority residing in low-income and middle-income countries. Globally, dementia entails significant economic burdens in 2019, amounting to a cost of 1.3 trillion US dollars. Informal caregivers devote considerable hours to providing care for those affected. Dementia imposes a greater caregiving and disability-adjusted life-year burden on women. A recent study has established a correlation between prolonged Proton Pump Inhibitor (PPI) usage and dementia, in addition to other neurodegenerative conditions. PPIs are frequently prescribed to treat peptic ulcers and GERD (gastroesophageal reflux disease) by decreasing stomach acid secretion. They alleviate acid-related symptoms through the inhibition of acid-secreting H^+^-K^+^ ATPase. In a number of observational studies, cognitive decline and dementia in the elderly have been linked to the use of PPIs. The precise mechanism underlying this relationship is unknown. These drugs might also alter the pH of brain cells, resulting in the accumulation of amyloid-beta (Aβ) peptides and the development of Alzheimer's disease (AD). Despite the compelling evidence supporting the association of PPIs with dementia, the results of studies remain inconsistent. The absence of a correlation between PPI use and cognitive decline in some studies emphasizes the need for additional research. Chronic PPI use can conceal underlying conditions, including cancer, celiac disease, vitamin B12 deficiency, and renal injury, highlighting dementia risk and the need for further investigations on cognitive health.

## 1 INTRODUCTION

Dementia, or the loss of cognitive function, is a major public health issue worldwide. Dementia is a cognition disorder characterized by significant challenges in autonomously carrying out daily activities. Dementia is most accurately characterized as a condition rather than an individual disorder [1]. As per the World Health Organization's report, the global prevalence of dementia exceed 55 million by 2023, with over 60 percent of those affected residing in low- and middle-income countries. Approximately 10 million new cases are reported annually [2]. The cognitive dysfunction arises from a variety of brain disorders and injuries. AD comprises 60-70% of all cases of dementia, making it the most prevalent form [2]. It ranks as the seventh most significant contributor to mortality and is a leading cause of disability and reliance among the geriatric population globally [3]. Dementia incurred a global financial burden of $1.3 trillion in 2019; informal caregivers supervised and provided care for affected individuals for an average of five hours daily [4].

Women are disproportionately impacted by cognitive impairments, both in direct and indirect ways. Women contribute 70% of dementia care hours and have a higher disability-adjusted life year and mortality rate associated with dementia [2, 5]. Although neurodegenerative diseases, such as AD are widely recognized as the main etiological factors contributing to dementia, recent research has provided insights into a possible association between PPI usage and the onset of dementia [6]. PPIs are a class of drugs that are frequently prescribed to inhibit the production of gastric acid. They are extensively used to treat GERD, peptic ulcer, and other acid-related disorders [7].

PPIs are the most commonly used and sold medications worldwide. By inhibiting the enzyme H^+^, K^+^ ATPase (proton pump), which is the final stage in the acid-release process, PPIs alleviate the symptoms linked to an overabundance of acid production [8]. The principal role of the proton pump is to facilitate the movement of H^+^ ions from the cytoplasm to the secretory canaliculus of the parietal cells. Additionally, it supports the return of K^+^ ions from the stomach lumen to the parietal cells [7, 9]. Recent years have seen increased concerns regarding the potential adverse effects of long-term PPI use, such as an elevated risk of developing dementia [10].

On the other hand, a study found that routine use of PPI increases the risk of all-cause dementia, comprising 932 cases of AD and 524 cases of vascular dementia. PPI users had a higher prevalence rate of all-cause dementia events per thousands of individuals compared to non-users. The research also discovered a strong link between the APOE ε4 genotype and the use of PPIs for dementia. This link was significant in people who were heterozygotes for APOE ε4 [11, 12].

According to a study, a final selection of six cohort studies was made by Comparing PPI users with non-PPI users for dementia and AD, respectively. The meta-analysis did not find a statistically significant correlation between PPI usage and a higher risk of dementia [11]. From the last sixteen years, extensive, multi-center health data revealed that individuals prescribed a PPI had a lower incidence of dementia compared to those who were not. The study used Wales' Secure Anonymized Information Linkage Databank data, excluding those with delirium codes, minor cognitive impairments, or dementia histories. The study also found that drugs used in conjunction with comorbidities, such as head injuries, were confounding factors [13]. However, the exact mechanism that governs this correlation is still unknown and is currently the focus of ongoing scientific investigation. Masters *et al.* (1985) identified Aβ peptides as pivotal in the pathogenesis of AD, the most prevalent form of dementia [14]. Studies have indicated that administering PPIs could disrupt the pH equilibrium in brain cells, facilitating the aggregation and buildup of Aβ peptides. This, in turn, could result in neurotoxicity and cognitive decline [6].

Notwithstanding these captivating discoveries, it is critical to acknowledge that the body of evidence concerning the correlation between the use of PPIs and dementia is ongoing to develop, and various studies have documented contradictory findings [11-13]. Furthermore, the study that looked at electronic health records from the Secure Anonymized Information Linkage (SAIL) Databank in Wales from 1999 to 2015 found no proof that using PPIs raises the risk of dementia. The study compared 183,968 PPI users with 3,765,744 non-users. Possible confounding factors include preexisting conditions like cardiovascular disease and depression, as well as drugs used to treat these conditions, which could explain the observed association with dementia. The study suggests that PPI usage may not be a significant risk factor [12]. Since some studies have not found a significant association between PPI usage and cognitive decline or dementia, more studies need to be conducted to establish a conclusive causal relationship between the two [12, 14]. Prolonged administration of PPIs has been associated with a range of safety concerns, including but not limited to cancer [15, 16], the hiding of symptoms associated with underlying medical conditions [17], celiac disease [18], deficiency in vitamin B12 [19], modifications in renal function [20], and dementia [21, 22]. The study analyzed 73,679 individuals aged 75 or older without dementia. Patients receiving regular PPI medication had a significantly higher risk of incident dementia compared to those not receiving medication (n = 2950; mean age = 83.8; 77.9% female) [21].

In addition to critically evaluating the current evidence on PPI-associated dementia, this article provides a synopsis of the studies conducted thus far, along with their methodologies and results. We intend to contribute to a comprehensive comprehension of this crucial and detailed matter by analyzing the merits and drawbacks of the existing literature.

## 2 POSSIBLE PATHOLOGICAL MECHANISM INVOLVED IN PPI-LINKED DEMENTIA

### 2.1 The Complex Relationship Between PPIs and Dementia

Prescriptions for PPIs medications are predominantly for acid-related gastrointestinal disorders, peptic ulcers, and GERD [9]. Although these drugs are generally well-tolerated, their potential association with dementia and Aβ plaques has piqued the interest of researchers [23, 24]. Aβ plaques are aberrant accumulations of Aβ protein fragments within the brains of individuals with AD. Plaques are widely recognized as a defining characteristic of the disease and are found to play a role in the dementia progression [25, 26].

Multiple studies have examined the possibility of a correlation between PPI use and dementia. Although preliminary research indicated a potential correlation between prolonged PPI use and an elevated likelihood of developing dementia, more recent inquiries have yielded inconclusive findings [11-13]. Furthermore, several studies have reported a marginally elevated risk or no association [6, 27, 28]. In a prospective population-based trial, 827 patients (23.7%) experienced dementia after a mean follow-up of 7.5 years, with 670 exhibiting probable or potential AD. PPI exposure did not show a link to dementia risk [27]. Notably, causality cannot be established through observational studies, which rely on data from actual patient populations and have inherent limitations. Additional factors may influence the observed associations, such as the underlying medical conditions treated with PPIs [29, 30].

The precise mechanism of PPIs affecting Aβ plaques or dementia remains incompletely elucidated. Extracellular deposition of Aβ plaques, which induce oxidative and inflammatory harm to the brain, is a prominent feature of AD [14]. These Aβ species are generated through the cleavage of amyloid precursor protein (APP) by β-secretase and γ-secretase (also referred to as β-site APP-cleaving enzyme 1 [BACE^1^]) [31].

Badiola *et al.* (2013) discovered that PPIs elevated levels of Aβ in rodents' brains through their effect on β and γ-secretase enzymes [23, 32]. Additionally, PPIs can increase BACE1 activity, leading to increased production of Aβ37 and Aβ40. Alzheimer's associated dementia is thought to be most frequently associated with the pathogenic species Aβ42, while Aβ40 is the most frequently synthesized subtype [14, 24]. In light of the potential hazards associated with Clostridium difficile infection and bone loss, the American Geriatrics Society (AGS) 2015 updated beers criteria advises against the regular administration of PPIs for longer than eight weeks in the elderly, except for high-risk patients [33]. START/STOPP criteria recommend discontinuing or reducing PPI therapy in older patients with erosive esophagitis or uncomplicated peptic ulcer disease who have been on the medication for more than eight weeks [34].

In summary, although scholars have shown interest in investigating the possible correlation between the use of PPIs and dementia, the available evidence is still ambiguous. Certain studies propose a marginally elevated risk, whereas others fail to identify any substantial correlation. It is imperative to acknowledge that observational studies possess inherent limitations in that they cannot establish causality. Many factors, such as individual patient characteristics and underlying medical conditions, could account for the observed associations. PPIs may influence Aβ plaques or dementia through an unidentified mechanism; therefore, additional research is required to elucidate this association.

### 2.2 Neurological Implications of Prolonged PPI Use

Life-sustaining nutrients and oxygen are transported to every tissue and organ in the body *via* the blood vessels. As a result of the Blood-Brain Barrier (BBB), a unique characteristic of the blood vessels that supply the Central Nervous System (CNS) with blood, the passage of ions, molecules, and cells between the blood and the brain is strictly regulated. Deviations from these barrier properties are pivotal in the pathogenesis and advancement of numerous neurological disorders, given that they support healthy neuronal function and protect brain tissue from pathogens and toxins [35].

The estimation of PPI penetration across the BBB was based on a study. Ortiz-Guerrero *et al.* (2018) determined the area under the curve (AUC) of concentration versus time in the brain of male Sprague-Dawley rats by dividing the AUC in the blood by the AUC following intravenous (IV) administration of 10 mg/kg omeprazole. Furthermore, with a blood-to-brain distribution coefficient of 0.15, a single intravenous dose of omeprazole can reach the central nervous system, suggesting that either chronic or short-term use may impair cognitive performance [6]. Moreover, *in vivo* and *in vitro* pharmacokinetic studies indicate that lansoprazole may traverse the BBB [6, 36].

Chronic use of PPIs may result in neurological side effects associated with direct or indirect systemic complications (*e.g.,* magnesium and vitamin B12 deficiency) or effects on neurons after the drugs cross the blood-brain barrier [37]. Diverse PPIs, including lansoprazole, esomeprazole, and pantoprazole, have been associated with neurological adverse effects such as vertigo and migraines. Adverse effects affecting the CNS are documented less frequently; they include delirium, depression, diplopia, restless sleep, drowsiness, insomnia, nervousness, tremors, sensory and perceptual anomalies, including hallucinations, and delirium [38]. Drawing from the available substantial evidence, it can be inferred that prolonged administration of PPIs may give rise to neurological adverse effects. As a result, long-term PPI therapy should be approached with caution, given its potential implications for cognitive function and systemic effects Fig. (**1**).

### 2.3 The Gut-Brain Axis, PPIs, and the Microbiota: Implications for Cognitive Function and Neurodegenerative Risk

The emotional and cognitive regions of the brain are linked to the peripheral functions of the intestine *via* the gut-brain axis, which facilitates bidirectional communication between the enteric and central nervous systems [39]. Many microorganisms, including the human host, inhabit the intricate ecosystem called the intestinal microbiota. The resident gut bacteria play a critical role in numerous physiological processes, such as sustaining the integrity of the gut mucosal barrier, facilitating energy production, defending against pathogens, and modulating the immune system. Bacterial communities of different densities and compositions congregate in each segment of the digestive tract [40].

In a recent scientific advancement, the importance of the intestinal microbiota in influencing these associations has been brought to the forefront. The relationship between microbiota and the gut-brain axis appears bidirectional, as neurological, endocrine, immune, and humoral connections transmit signals from the brain to the gut microbiota and vice versa [41]. Studies have indicated that PPIs have the potential to induce neurodegenerative disorders, such as dementia, through their impact on the brain-microbiota axis [42]. Long-term use of PPIs alters the microbiota of the small intestine, where Small Intestinal Bacterial Overgrowth (SIBO) can develop as the “defense barrier” afforded by gastric acid is eliminated [43]. SIBO is a pathological condition distinguished by an overabundance or aberration of bacterial species inhabiting the small intestine. SIBO is characterized by an intestinal count of over 105 bacteria per milliliter of upper intestinal aspirate. The typical value obtained from upper intestine aspirate is below 104 per milliliter [44, 45].

Inflammation-induced by bacterial products, such as microbiome-associated metabolites, in the systemic circulation may exacerbate dementia risk [46]. While there have been divergent findings regarding the association between dementia and the gut microbiome, the precise mechanism by which the gut microbiome and its associated metabolites impact cognitive function remains unknown. For example, studies have documented that individuals diagnosed with dementia exhibited variations in Bacteroides levels, ranging from reduced to elevated [47-49]. In more severe cases, patients may experience malnutrition, hepatic alterations, rosacea, joint pain, anemia, tetany in hypocalcemia induced by vitamin D3 deficiency, metabolic bone disease, and polyneuropathy due to vitamin B12 deficiency [50]. Prolonged use of PPIs can induce intestinal dysbiosis, thereby increasing the susceptibility to infection and intestinal disorders. An additional study has established a correlation between changes in the composition of the gastrointestinal microbiome and dementia [51]. Recently, there has been a proposition to augment the efficacy of PPI medication by incorporating probiotic nutrition. In addition to augmenting the therapeutic effects of PPIs, probiotic supplementation may decrease intestinal dysbiosis and the adverse effects of long-term PPI use [52].

16S rRNA gene sequences were utilized by Imhann *et al.* (2016) to examine the intestinal microbiota of 1815 individuals who had taken PPIs. The relative abundances of 20% of bacterial taxa, including species of *Escherichia coli* and the genera *Staphylococcus, Streptococcus*, and *Enterococcus*, were significantly higher in PPI users than non-users [53].

According to a study by Tsuda *et al.* (2015), the composition of microorganisms in the gastric fluid microbiomes of PPI users and non-users was comparable. When PPIs were discontinued, however, the beta diversity of the microbiota in the gastric fluid increased significantly. The administration of PPI elevates the pH levels in the upper gastrointestinal tract and stomach, facilitating the passage of bacteria, especially detrimental ones, from the stomach to the intestines [54]. Imhann *et al.* (2016) found that the gut microbiomes of PPI users are altered, containing a more significant number of microbes than are typically observed in the mouth and throat. Additionally, the researchers identified that the utilization of PPIs induces notable modifications to the gastrointestinal microbiome, including a surge in the abundance of the bacterial genera *Enterococcus, Gammaproteobacteria*, and *Enterobacteriaceae*. Elevated concentrations of the bacteria Clostridium difficile have been associated with infections in both human and animal models [55].

To summarize, the complex interaction between the gut-brain axis and the gut microbiota significantly impacts cognitive function, among other physiological processes. By interfering with the integrity of the gastric acid-small intestine barrier, prolonged PPI use may modify the small intestine's microbiota, potentially exacerbating conditions such as SIBO. Research has indicated that there may be a correlation between dysbiosis induced by PPIs and neurodegenerative diseases, such as dementia. This finding emphasizes the criticality of contemplating probiotic supplementation to alleviate these consequences. Furthermore, scholarly investigations suggest that long-term use of PPIs may substantially influence the diversity and composition of the intestinal microbiota, thereby promoting the growth of detrimental bacteria. This underscores the critical nature of taking their long-term usage into account.

### 2.4 PPI-Induced Micronutrient Deficiencies and Their Implications for Cognitive Health

Using PPIs may reduce the absorption of specific micronutrients, including magnesium and vitamin B12. Cognitive impairment has been linked to deficiencies in the nutrients mentioned above. Evidence suggests that using PPI may moderate the risk of hip fracture, as indicated by an RR = 1.26 [56]. The absorption deficiencies most extensively studied concerning chronic PPI administration are β-carotene, zinc, vitamin C, hypomagnesemia, vitamin B12, and iron deficiency anemia [57-59]. According to the study, vitamin B12 deficiency cannot be cured in individuals who consume a nutritious diet and take the recommended PPI dosage. Furthermore, the micronutrient deficiency is associated with a two to fourfold increased risk in individuals aged 40 and above and chronic nutritional issues [25].

Moreover, PPI medication has significantly improved iron malabsorption by increasing the risk of achlorhydria due to gastric acid hyposecretion. Dietary iron comprises either non-heme iron (66 percent) or heme iron (22 percent). Gastric acid significantly enhances the absorption of non-heme iron by facilitating the dissolution and solubilization of iron ions in non-heme-containing foods and forming complexes with carbohydrates and amines [60, 61]. Hypomagnesemia has the potential to induce neuromuscular complications (*e.g.,* tetany and convulsions), cardiac complications (primarily arrhythmias), osteoparathyroidism, osteomalacia (likely attributable to vitamin D deficiency), and osteoporosis [62]. According to recent research, PPI is associated with reduced magnesium levels [63, 64]. Although generally accepted for their efficacy and tolerability, prolonged administration of PPI has been linked to adverse effects, such as impaired calcium absorption. Due to the pH dependence of calcium absorption, a hypothesis has been developed that hypocalcemia induced by PPIs is associated with reduced calcium bioavailability in gastric achlorhydria [65]. The possibility of micronutrient changes affecting the mineral balance of the bone in long-term PPI consumers is confirmed by one study. PPI users had decreased blood levels of vitamin D, and vitamin D deficiency was more prevalent in this group. Lastly, zinc deficiency did not exhibit a statistically significant trend among PPI users [66]. A putative neuroprotective effect was observed for each of the micronutrients in question [67]. An additional functional attribute of PPI-induced increase in Aβ levels could be the regulation of Aβ degradation by microglial lysosomes [21]. Aβ deposits stimulate microglia, innate immune cells in the brain, resulting in an inflammatory response in the central nervous system [68]. Concluding these noteworthy discoveries, it is possible that prolonged use of PPIs could result in a deficiency of micronutrients such as vitamin D, magnesium, iron, calcium, and B12, all of which possess potential neuroprotective properties. This emphasizes the criticality of monitoring and controlling nutrient levels in PPI users over an extended period of time in order to mitigate any potential cognitive consequences Fig. (**2**).

## 3 PHARMACOKINETICS AND PHARMACODYNAMICS OF PPI: INSIGHTS INTO TARGETED INHIBITION OF H^+^/K^+^-ATPase AND NEUROLOGICAL IMPLICATIONS

PPIs are very selective drugs that work by blocking the H^+^/K^+^-ATPase proton pump. This stops the production of acid in the parietal cells of the stomach. They modulate acid secretion at the molecular level, making them ideal for managing conditions with excessive gastric acid production [7]. These drugs have complicated pharmacokinetics and pharmacodynamics profiles, including activating binding and then, activating, binding, and inhibiting the stomach H^+^, and K^+^-ATPase enzymes. Omeprazole, pantoprazole, lansoprazole, and rabeprazole are prodrugs that are activated by acid. Once activated, they form a disulfide bond and covalently attach to the gastric H^+^, K^+^-ATPase, mainly at the Cys813 site. This binding inhibits the proton pump enzyme, stopping gastric acid secretion. These PPIs have similar basic compounds, such as pyridine and benzimidazole, but their ADME and pharmacodynamics differ slightly [69].

To comprehend the pharmacodynamics of PPIs, one must take into account various factors, including PPI accumulation in parietal cells, the proportion of the pump enzyme at the canaliculus, the *de novo* synthesis of a new pump enzyme, PPI metabolism, the stability of PPI binding, and the covalent binding of PPI in the parietal cell. The intragastric pH profile and the area under the plasmic concentration curve are crucial markers of PPI efficacy. PPIs have an elimination half-life of approximately one hour. Cytochrome P450 enzymes, particularly CYP2C19 and CYP3A4 polymorphisms, are involved in the metabolism of PPIs. Depending on the CYP2C19 genotype status, the racemic mixing of PPIs shows pharmacokinetic and pharmacodynamic variations. R-lansoprazole boosts medication activity, while S-omeprazole's resistance to CYP2C19 improves intragastric pH regulation. Among the PPIs now on the market, delayed-release formulations, such as dexlansoprazole have demonstrated better control over intragastric pH [69, 70]. The heterodimeric enzyme gastric H^+^, K^+^-ATPase, composed of α and β subunits, is primarily targeted by PPIs. The activation (A), phosphorylation (P), and nucleotide-binding (N) domains make up the catalytic sites in the α subunit. During the proton-pumping process, the α-β heterodimeric enzyme, specifically the α subunit, experiences conformational changes [7]. PPIs, being acid-activated prodrugs, transform into sulfenic acids or sulfenamides, making covalent connections with cysteines accessible from the luminal surface of the ATPase. Because of this covalent binding, these drugs have inhibitory effects that last far longer than their plasma half-life. The drug's brief blood half-life and the requirement for acid activation may limit effectiveness, especially during nighttime acid suppression [69]. The elimination half-life of all PPIs is approximately an hour; however, due to variations in drug formulation and/or dietary effects, the time to maximum plasma concentration (tmax) might vary significantly from one hour to five hours. ATP12A/ATP1AL1, one of the genes encoding H^+^/K^+^-ATPase, is expressed in the human brain, while ATP4A is limited to gastric epithelium cells [69, 71]. It has been shown that the CNS exhibits H^+^/K^+^-ATPase activity [72], which affects K^+^ and acid/base balance [20]. H^+^-ATPases and V-type ATPases, two vesicular proton pumps, are crucial for the storage of neurotransmitters in synaptic vesicles. In addition to these H^+^-ATPases, vesicular H^+^/K^+^-ATPases appear to be crucial for nerve-ending exo- and endocytosis [73, 74].

The capacity of PPIs to cross the BBB is an important pharmacokinetic feature of these drugs. One study found that 15% of an intravenous dosage of omeprazole reached the central nervous system [75]. Lansoprazole has also been found to cross the blood-brain barrier [36]. In general, PPIs like lansoprazole, esomeprazole, and pantoprazole appear to cross the BBB, which is consistent with the presence of adverse neurological effects such as headache, dizziness, diplopia, depression, difficulty sleeping, fatigue, tremor, delirium, anxiety, and hallucinations [37, 38, 76-79].

In conclusion, PPIs show their therapeutic effect by selectively blocking the H^+^/K^+^-ATPase proton pump, disrupting the final step of gastric acid production. Even though their pharmacokinetics and pharmacodynamics are complicated, involving drug activation, binding, and enzyme inhibition, PPIs have made treating acid-related disorders much easier, improving people's health-related quality of life overall. Also, the fact that PPIs can cross the blood-brain barrier brings up pharmacokinetic issues. These issues could lead to neurological side effects and systemic changes, which stresses the importance of close monitoring during clinical use.

## 4 DIFFERENT TYPES OF DEMENTIA AND THEIR SUSCEPTIBILITY TO PPIs

Although the term dementia refers to a clinical condition of gradual cognitive deterioration, its subcategories are categorized based on the source of dementia. The four most prevalent forms of dementia are frontotemporal, Lewy body, vascular, and AD [80].

### 4.1 Alzheimer’s-Type Dementia

AD stands as the most prevalent neurodegenerative disease leading to dementia, encompassing 60% to 80% of cases. The accumulation of Aβ plaques and neurofibrillary tangles, initially in the entorhinal cortex and hippocampus, results in neuronal injury and subsequent death, linking it to the etiology of AD [81]. Memory and thinking skills get worse because cholinergic neurotransmission decreases, mainly because choline acetyltransferase activity decreases and the number of cholinergic neurons decreases. As the pathology progresses to other brain regions, a cascade of neuronal death exacerbates AD symptoms. Genetic factors, including PSEN1, APP, and PSEN2 mutations for early-onset AD and Apolipoprotein E for late-onset AD, play a role in AD development. While late-onset AD typically occurs after the age of 60, early-onset AD is associated with specific genetic mutations in individuals aged 30 to 60 [82]. Six cohort studies found no significant correlation between PPI usage and an increased risk of dementia or AD. The pooled relative ratio for dementia was 1.23 compared to those without PPIs, as 1.23 compared to those without PPIs, and for AD, it was 1.01. The study observed a follow-up period of 0.67 to 9 years [11].

### 4.2 Vascular Dementia (VD)

VD, constituting the second most prevalent form of dementia (20%), is also known as multi-infarct dementia. It arises from neuronal oxygen deprivation due to conditions obstructing or reducing blood flow to the brain, with stroke being the primary cause [80]. The symptoms of vascular dementia vary depending on the affected brain regions and the severity of blood vessel damage. Significant stroke aftermaths include confusion, disorientation, difficulty in speech and comprehension, and vision loss. Memory may remain unaffected, but abrupt changes in executive function may emerge. Conversely, multiple small strokes lead to a gradual decline in executive function as cumulative damage occurs. Mixed dementia refers to the simultaneous presence of AD and vascular dementia [83, 84].

### 4.3 Lewy Body Dementia (LBD)

Aberrant deposits of the alpha-synuclein protein, known as Lewy bodies, localized within neurons, attributed to LBD. About 5 to 15 percent of people with dementia have LBD. It is marked by apparent symptoms like frequent, complicated visual hallucinations, sudden tremors, and changing cognitive impairment that show up as changes in attention and alertness [85]. Initial phases manifest rigidity, bradykinesia, and REM sleep disorders, while advanced stages often exhibit prevalent memory loss. LBD contrasts with Parkinson's Disease Dementia (PDD) based on temporal progression and clinical manifestations, where tremor is more characteristic in PDD, while postural instability and gait difficulties predominate in LBD [86].

### 4.4 Frontotemporal Dementia (FTD)

FTD is a broad name for conditions affecting the frontal and temporal lobes of the brain, such as Pick's disease. Compared to AD, this type of dementia often manifests at an earlier age (40-75 years). Key characteristics of FTD include behavioural abnormalities and personality changes that start early in the illness. The visuospatial function is typically unaffected, in contrast to AD. Frontotemporal lobar degeneration (FTLD), linked to a variety of diverse diseases, is the cause of these alterations. The diagnosis of FTD is still difficult despite recent improvements in its characterization; some individuals are written off as normal, while others may receive an incorrect diagnosis of mental illness or AD [87].

In a study by Imfeld *et al.*, individuals over 65 using PPIs for an extended period showed no elevated risk of developing VD or AD compared to non-users (adjusted odds ratio [OR] 0.85 and 0.90, respectively), regardless of whether the PPIs were used singly or in combination [88]. Similarly, Taipale *et al.* observed, with a 3-year lag window, that higher doses (≥1.5 defined daily doses per day) and longer duration of PPI use (≥ three years) did not result in an increased risk of Alzheimer's dementia (OR 1.03, 95% CI 0.92-1.14, and OR 0.99, 95% CI 0.94-1.04, respectively) compared to non-use [89]. Four European observational studies have looked into the relationship between PPI use and dementia, based on a 2017 systematic review. Three studies have positively linked omeprazole, esomeprazole, lansoprazole, and pantropazole to dementia. Cohorts taking PPIs had a roughly 1.4-fold higher risk of developing dementia overall (95% CI, 1.36-1.52; *P* < 0.001) [90]. Likewise, a link between PPI use and dementia has been discovered in recent prospective cohort research in the Asian population (n = 15726, 7863 PPI users) (HR, 1.22; 95% confidence range, 1.05-1.42) [14]. On the other hand, the systematic review's fourth European study discovered a negative correlation (OR dementia with PPI use = 0.94 (95% CI, 0.90-0.97), *P* = 0.0008) [91].

## 5 EXERCISING CAUTION IN PPI PRESCRIPTIONS: ADDRESSING THE POTENTIAL CORRELATION WITH DEMENTIA

Given the potential correlation between the use of PPI and dementia, medical professionals must exercise prudence when prescribing these drugs, especially to geriatric patients or for extended periods [10]. It is imperative to conduct regular reassessment and risk-benefit analysis of continued PPI therapy. Healthcare providers are concerned about the potential correlation that may exist between the use of PPIs and dementia [12]. The potential correlation between the use of PPIs and dementia has significant clinical ramifications, underscoring the importance of exercising prudence when prescribing these drugs, particularly for extended periods of time or in geriatric patients.

### 5.1 Balancing Risk and Benefit: Individualized Decision-Making for PPI Therapy

Physicians must evaluate the risks and benefits individually for each patient when prescribing PPIs. When considering PPI therapy, it is important to evaluate the potential risk of developing dementia against the benefits that include symptom relief and an enhanced quality of life [92]. In this evaluation, the severity of the patient's acid-related disorder is crucial. In the case of patients afflicted with severe symptoms or conditions, such as Barrett's esophagus or erosive esophagitis, the potential advantages of PPI therapy in terms of symptom alleviation, healing promotion, and complication prevention might surpass the possible drawbacks [93]. In conclusion, the risk-benefit evaluation must be personalized for every patient, considering their unique clinical conditions, the gravity of their acid-related disorder, and the accessibility of alternative therapeutic alternatives. A comprehensive assessment of the potential advantages and disadvantages, a collaborative decision-making methodology involving the patient, and periodic reassessment are all necessary components of this procedure to guarantee that the benefits of PPI therapy persistently surpass the risks in the long run.

### 5.2 Age-Related Metabolic Changes and the Controversy Surrounding PPI-Associated Dementia

Alterations in metabolism and a decline in the function of numerous cells and tissues are common physiological changes accompanying aging. These changes can significantly impact the overall health and well-being of older people. The medical community has devoted considerable attention to this phenomenon due to its far-reaching implications for numerous facets of geriatric care [94, 95]. Hormonal fluctuations, decreased physical activity, and alterations in body composition cause age-related fluctuations in the metabolism of individuals. These metabolic alterations may all impact dietary absorption, energy expenditure, and metabolic rate [96]. Age-related metabolic changes have been linked to a higher incidence of cardiovascular disease, metabolic syndrome, and insulin resistance in the elderly [97].

A study using a computerized neuropsychological test battery on 13,864 participants in the Nurses' Health study found no significant association between PPI use and cognitive performance, indicating a weak or noticeable relationship between these variables in the studied population [98].

### 5.3 Elderly Vulnerability to PPI-Related Health Concerns and Dementia

The susceptibility of elderly patients to the adverse effects linked to PPIs may be increased. The potential negative consequences encompass a range of health issues, such as kidney dysfunction, osteoporotic fractures, gastrointestinal infections, mineral and vitamin B12 deficiencies, and, dementia [22, 99, 100]. Age-related variables, including reduced gastric acid secretion, compromised bone health, and impaired nutrient absorption, may contribute to this population's increased susceptibility [101]. Multiple case-control studies have demonstrated that using PPIs increases the risk of bone fracture in older people. Due to contradictory findings in the published research, it is unknown at what dosage and how long PPI therapy increases the risk of osteoporotic fractures. Researchers in Taiwan employed case-control studies utilizing a health insurance database and daily doses (DDDs) defined by the World Health Organization; higher DDDs indicated greater exposure to PPI medication [33, 102]. There may be an increased susceptibility to cognitive impairment among older people.

Further, a meta-analysis study concluded that there was no significant correlation between the use of PPIs and the risk of dementia or AD [11]. Given these age-related variables and potential hazards, medical professionals encounter the intricate challenge of reconciling the advantages of PPI treatment with the possible negative consequences, particularly in the elderly population. Personalized treatment plans, systematic monitoring for adverse effects, and thoughtful evaluation of alternative management approaches are integral elements in delivering comprehensive healthcare to this susceptible demographic. Although the precise mechanisms underlying the association between PPIs and cognitive decline remain obscure, it is imperative that healthcare providers closely assess the necessity of long-term PPI use in elderly patients, especially those with preexisting cognitive impairment, in light of this risk.

### 5.4 Polypharmacy and Medication Interactions in the Elderly

In order to increase efficacy and control co-morbid conditions, concurrent use of multiple medications is frequently advised. Polypharmacy refers to the practice of simultaneously ingesting five or more medications [103]. Many geriatric syndromes, elevated healthcare expenditures, heightened likelihood of adverse drug events and drug-drug interactions, non-compliance with medication regimens, and increased financial burdens have all been associated with polypharmacy [104]. The concurrent use of numerous medications by elderly patients increases the probability of polypharmacy and drug interactions. Healthcare professionals should exercise caution when evaluating the medication regimen of elderly patients, taking into account possible drug-drug interactions and making necessary modifications to the treatment strategy [105]. Due to their impact on gastric pH and their capacity to obstruct particular liver enzymes implicated in drug metabolism, PPIs may interact with several medications [106]. For instance, PPI has the potential to impede the absorption of specific medications, including antiretroviral drugs like Atazanavir and antifungal agents such as ketoconazole and itraconazole, which are optimally absorbed in an acidic environment. Potentially causing adverse effects or altering the efficacy of pharmaceuticals metabolized by cytochrome P450 enzymes are PPI interactions [107]. According to a study, researchers have raised concerns about the use of PPIs in cancer treatments due to potential interactions with tyrosine kinase inhibitors and immune checkpoint inhibitors, as well as systemic chemotherapy medications like 5-FU or capecitabine, methotrexate, and pemetrexed. This issue affects medical oncologists daily and suggests a moratorium on PPI use in cancer patients undergoing treatment, as it poses a high risk of interaction and lacks benefit for treating cancer symptoms [108].

Scientific investigation has primarily focused on understanding the interaction profiles between pantoprazole sodium and omeprazole. Omeprazole has an intermediate affinity for the CYP3A4 enzyme and a high affinity for CYP2C19, making it a pharmaceutical agent with a high risk of drug interactions. Pantoprazole sodium, on the other hand, shows a reduced risk of drug interactions. However, lansoprazole, rabeprazole, esomeprazole, and dexlansoprazole have been studied to a lesser extent, resulting in fewer interaction profiles and weaker drug interaction potential [106, 109].

In order to identify possible drug interactions with PPIs or other substances, healthcare providers are obligated to conduct a comprehensive assessment of the medication regimen of elderly patients, which includes over-the-counter medications, botanical supplements, and vitamins [110]. Additionally, they must customize the treatment regimen for every patient, considering their unique medical circumstances, general well-being, and possible drug interactions. Modifying the dosage, scheduling, or selection of medications may be necessary to mitigate the potential for adverse interactions. Alternative treatment modalities without substantial drug interactions may be considered [111].

Based on the abovementioned findings, healthcare professionals and older people must communicate frequently and transparently. Patients must disclose to their healthcare provider all medications they currently utilize, encompassing prescription and over-the-counter products, to facilitate a comprehensive medication review and reduce the likelihood of drug interactions. Medication reconciliation is essential to identify and manage potential drug interactions. It entails comparing the medications a patient takes and the records maintained by their healthcare provider.

### 5.5 Personalized Approach to PPI Prescriptions for Elderly Patients

An individualized approach should be employed when prescribing PPIs to elderly patients, considering factors such as the patient's cognitive function, co-morbidities, overall health, and care objectives [112]. A comprehensive evaluation of the patient's health status ought to be conducted by healthcare providers, encompassing latent medical conditions and co-morbidities. Metabolic and renal dysfunction can potentially impact eliminating medications, including PPIs. Alternative treatment modalities might be considered [113].

Cognitive function evaluation is of the utmost importance in light of the potential association between PPI use and cognitive impairment. AD or cognitive impairment patients may be particularly susceptible to adverse effects. The investigation was unable to establish a link between PPI usage and an increased risk of dementia using extensive, multi-center health data [12]. Medical personnel should thoroughly evaluate PPI medication's potential benefits and drawbacks in such circumstances and, if required, explore alternative treatment approaches [92]. Healthcare providers must investigate and promote lifestyle modifications that support smoking cessation, weight management, dietary adjustments, and avoiding trigger foods. These measures may aid in symptom management and decrease PPI dependence [114]. When applicable, alternative treatment options might be contemplated. Acid-related conditions may be effectively managed with H2 blockers, such as ranitidine or famotidine, which may be associated with a lower risk of adverse effects than PPIs. Antacids, characterized by their ability to neutralize stomach acid and offer transient respite, might also be contemplated for mild or sporadic symptoms [115].

By adopting such personalized strategies, medical professionals can customize the course of treatment to suit every geriatric patient's unique requirements and situations. This process entails thoroughly evaluating alternative treatment alternatives, adjustments to the patient's lifestyle, and transparent communication with the patient's caretakers. This validates that the advantages of PPI therapy surpass the disadvantages and are consistent with the patient's holistic health objectives.

### 5.6 Routinely Monitoring, Evaluation and Reassessment of PPI Therapy in Elderly Patients

Regular monitoring and evaluation of elderly patients treated with PPIs is crucial for their treatment. This includes assessing their therapeutic response, determining if continued use is necessary, and monitoring for potential adverse effects. Consistent follow-up appointments allow healthcare providers to modify the treatment regimen based on the patient's evolving circumstances [33]. Some patients may persist in using PPIs beyond the initial resolution, potentially leading to unwarranted long-term usage [116]. Many elderly patients persist in taking PPIs for extended periods, requiring consistent monitoring to determine the necessity of ongoing use or the viability of alternative treatment modalities [117].

Monitoring for the potential adverse effects is essential to safeguard the patient's health. Individual characteristics such as age, kidney function, and the severity of the underlying condition may influence the optimal dosage of PPIs. Consistent follow-up appointments afford the chance to modify the dosage as necessary [33, 118].

Elderly patients may have concerns regarding their medications; addressing them during consultations can improve medication adherence and outcomes. During follow-up visits, it is critical to thoroughly examine the patient's complete medication regimen in order to detect any possible drug interactions or redundant medications [119]. Monitoring magnesium levels in individuals who have recently undergone a kidney transplant is especially important. Although monitoring of such micronutrients is controversial, it may be necessary for certain patients prescribed PPIs for prolonged durations [120, 121].

Furthermore, Continuous PPI therapy should be reassessed routinely to ascertain its continued necessity. After the patient's symptoms have resolved or improved, the dosage should be decreased progressively, or the medication should be discontinued. Consistent follow-up appointments can facilitate the assessment of the patient's therapeutic response and the detecting of potential adverse effects. Consistent follow-up consultations with the medical professional are crucial to assessing the patient's PPI treatment progress [115, 116]. The healthcare provider can evaluate the patient's symptoms, perform any required examinations, and ascertain the continued necessity of the medication during these scheduled appointments. If the patient's symptoms have substantially improved, the healthcare provider might contemplate the option of cessation of the PPI or a progressive reduction in dosage [38]. It is essential to highlight that the cessation of PPI therapy should be carried out with the supervision of a healthcare professional. An abrupt cessation may result in rebound acid production and a potential resurgence of symptoms. It may be imperative to gradually reduce the medication dosage to mitigate the potential adverse effects [122].

Based on the findings, healthcare providers must regularly monitor and evaluate elderly patients undergoing PPI treatment to ensure optimal outcomes. Healthcare providers should assess therapeutic responses, consider the need for continued use, and monitor potential adverse effects. Consistent follow-up appointments allow adjustments to treatment regimens, preventing unwarranted long-term usage. Reassessing the necessity of continuous therapy, decreasing dosage post-symptom resolution, and addressing individual concerns are essential measures to enhance medication adherence and minimize adverse effects.

### 5.7 Clinical Findings Showing the Association between PPI and Dementia Risk

A study involving 36,360 patients prescribed PPI medication between 2002 and 2015 found a modest increase in dementia risk but not a higher incidence of AD compared to 99,362 non-exposed participants. The study was based on CatSalut Database Data [123]. A retrospective cohort study found no significant difference in sequence ratio between dementia and PPIs (7342 pairings, sequence ratio 1.21), suggesting an overestimation of PPIs' link to dementia [124]. A study in Taiwan found that patients without a dementia history who started taking PPI (n = 6584) for about five years without a prescription were more likely to have dementia. The findings, based on insurance claims data from Taiwan, support the negative effects of PPIs on dementia risk, highlighting the need for better healthcare policies and interventions [125]. According to one more study that analyzed 23,656 veterans' records, including 11,828 PPI and non-PPI veterans. Dementia affected 9.5% of PPI veterans, while 6.3% of the control group experienced it. A significant correlation was found between PPI usage and dementia, with a 51.4% increase in the relative risk of dementia. PPI users had a 1.55% odds ratio for dementia risk compared to nonusers. Dementia rates varied among PPI users, with rabeprazole users having the highest rate at 12.8%, 10.9% for lansoprazole, 9.7% for omeprazole, 7.7% for esomeprazole, and 7.0% for pantoprazole [126].

A 5-year study found a correlation between PPI use and dementia in a retrospective cohort of 10,533 older people. The study observed three PPI use trajectories: short-term, intermittent, and long-term. Long-term and intermittent PPI users did not appear to have a higher risk of dementia compared to short-term users. Over a 4-year mean follow-up period, PPIs did not substantially increase the risk of dementia, regardless of the use pattern [127]. A study involving subjects with persistent cardiovascular and peripheral vascular disease found that using pantoprazole (40 mg daily, n = 8791) for three years did not cause any side effects, including cognitive impairment, in a placebo-controlled trial. The only possible side effect was an increased risk of gastrointestinal infections. Researchers conducted the study in a 3 × 2 partial factorial double-blind experiment [128].

A total of 7878 twins (n = 4314 for MADT and n = 3615 for LSADT) and 4821 participants had baseline data available for cross-sectional analysis and follow-up studies, respectively. When analyzing data from two large population-based studies, researchers could not find any correlation between PPI use and cognitive impairment [129]. A longitudinal clinical study found no link between PPI use and increased dementia or AD risk. The study relied on self-reported PPI use and a lack of dispensing data. In this study, a cohort of 50 volunteers was investigated, revealing that 884 individuals were regular consumers of PPI, 1,925 reported occasional PPI usage, and 7,677 participants had never taken PPI. At the initiation of the study, all subjects exhibited either normal cognitive function or mild cognitive impairment [130]. Among the 642,949 participants in the meta-analysis of 11 observational studies, women comprised 64%. The majority of the research only lasted between five and ten years. In all, 483,995 people did not utilize PPI, whereas 158,954 people did. The investigation did not support the suggested connection between PPI use and an elevated risk of dementia (Table **1**) [131].

## 6 CONSIDERING ALTERNATIVES TO PPI THERAPY FOR PATIENT SAFETY

In specific circumstances, alternative acid-suppressing medications, including antacids and H2 blockers (*e.g.,* ranitidine), may be considered viable substitutes for PPIs [132]. Concerning acetylcholinesterase inhibitors (AChEIs), donepezil is the best of the second generation. Donepezil (5-10 mg/kg) is now the standard treatment for AD and other forms of dementia in the elderly following the shutdown of tacrine production. There is a marked increase in the frequency of adverse events with the increase in dosage from 5 mg/d to 10 mg/d. Symptoms including rhinitis, nausea, vomiting, diarrhoea, and anorexia are among the adverse events reported in the higher dosage group [133, 134]. The FDA has authorized rivastigmine for the management of mild to severe AD. The recommended daily dosage is 6-12 mg/day. People using patches were less likely to suffer from specific side effects, such as nausea, vomiting, weight loss, and dizziness than those who used the capsules [135, 136].

Galantamine, a reversible AchE inhibitor and allosteric nicotinic receptor modulator, slows cognitive and functional loss in mild to severe Alzheimer 's-type dementia. The ADAS-cog score measures cognitive function. Galantamine 16 and 24 mg/day showed substantial improvements in patients with moderate AD. Placing a placebo on moderate AD patients resulted in a decrease, while 24 mg/day and 16 mg/day had no effect. Galantamine is generally considered safe and well-tolerated, with only 14% of participants stopping due to side effects in a meta-analysis of large placebo-controlled clinical trials. Common side effects include nausea (24%), vomiting (14%), diarrhoea (8%), stomach pain (8%), dyspepsia (8%), anorexia (10%), and weight loss (10%) due to dose-dependent cholinergic activation [137, 138]. In a randomized, double-blind, placebo-controlled trial, patients with Lewy-body-associated dementias were administered either 20 mg of memantine once daily (titrated in 5 mg increments over 4 weeks) or a placebo, maintaining a 1:1 ratio. The study suggests that memantine may benefit patients with Lewy-body-associated dementias, particularly in addressing behavioural symptoms. These findings align with prior randomized, placebo-controlled trials, contributing to a growing body of evidence. Gastrointestinal distress, affecting 10% of patients, is among the potential side effects, including symptoms such as constipation, nausea, and vomiting, along with other adverse effects like dizziness, confusion, headaches, hallucinations, aggression, pain, hypertension, and heart failure [139-141].

Acetyl-l-carnitine, a derivative of carnitine, is believed to help treat dementia diseases due to its ability to increase CSF concentrations after oral and intravenous administration. However, its positive effects on cognitive performance, dementia severity, functional capacity, and clinical global impression are not evident. Gastrointestinal side effects, such as diarrhea, nausea, and vomiting, are common. The drug's easy penetration into the blood-brain barrier is due to its ability to raise CSF concentrations. A single 500 mg dose produced a peak plasma concentration of 1.19 μg/mL [142-144]. According to a study, participants received a dose of 0.15 mg of huperzine A orally twice a day for six months. Huperzine A did not significantly improve the vascular dementia patient's condition. Among those adverse effects, the Huperzine A group saw a higher likelihood of moderate peripheral cholinergic side effects, such as diarrhoea, mild bellyache, and dry mouth, than the placebo group did [145, 146]. A double-blind, randomized, placebo-controlled experiment showed quetiapine, an antipsychotic, effectively reduced agitation and psychosis in dementia patients. Participants started with 25 mg daily and gradually increased to 100 mg daily by the 14^th^ day. Further, another research supports this finding, showing quetiapine's effectiveness in managing dementia-related agitation at a dose of 200 mg/day. However, adverse effects included sleepiness, vomiting, drowsiness, constipation, and gait changes. Traditionally, antipsychotics manage agitation and psychosis in dementia patients [147, 148].

Aducanumab, a monoclonal antibody designed to target Aβ aggregates, has received FDA approval for the treatment of moderate cases of AD. A randomised, double-blind, placebo-controlled study was used to examine Aducanumab's pharmacokinetics, safety profile, and tolerability in people with mild to moderate AD symptoms. The study found that doses below 30 mg/kg showed good tolerance with no severe adverse effects. However, at higher doses, the Aducanumab infusion showed a slight deceleration of cognitive decline in patients with early-onset AD dementia or mild cognitive impairment. The medication may induce amyloid-related anomalies in imaging, so individuals with AD should consider sparing use due to its mild cognitive effects [149, 150]. A double-blind 18-month trial involving participants aged 50-90 with early AD symptoms found that intravenous lecanemab, a medication, decreased amyloid markers in early AD and produced less severe cognitive impairment compared to a placebo. The treatment caused amyloid-related imaging abnormalities in 12.6% of subjects and infusion-related responses in 26.4%. Researchers reported cases of siderosis, cerebral macrohemorrhages, and cerebral microhemorrhages. The trial was double-blind and involved amyloid-positive PET scans or CSF tests [151, 152].

Brexpiprazole, a third-generation antipsychotic, has a stronger binding affinity to alpha1B, 5-HT2A, and 5-HT1A receptors than other third-generation antipsychotics. It has the potential to be a safe and effective treatment for agitation associated with AD dementia. However, patients taking 2 mg of brexpiprazole daily may experience headache, sleeplessness, dizziness, and urinary tract infection (UTI), with an incidence of ≥5% [153, 154]. Suvorexant is an orexin receptor antagonist that selectively blocks endogenous orexin neuropeptides that promote wakefulness at orexin receptors OX1R and OX2R, hence facilitating sleep. Suvorexant 10 mg (up to 20 mg depending on the clinical response) or placebo was administered in a double-blind, randomised, 4-week trial to individuals who satisfied the clinical diagnostic requirements for both probable AD dementia and insomnia. The most frequent side effect associated with the treatment drug was somnolence. Additional reports of diarrhoea, dry mouth, headaches, and falls were made in the patients [155, 156]. A population-based study linked exposure to PPIs to a higher incidence of all-cause dementia developing before the age of 90, regardless of when therapy was initiated based on the diagnosis. Higher risk estimations were produced by using PPIs for longer, cumulatively. The incidence of dementia increased with younger diagnostic ages [6].

In general, the clinical ramifications stemming from the possible correlation between the use of PPIs and dementia underscore the criticality of prescribing these medications based on informed judgment. Implementing risk-benefit analysis, reassessing treatment plans regularly, and exploring alternative treatment modalities are all essential to optimize therapeutic outcomes and ensure patient safety [11]. Healthcare providers must remain current on the most recent research and guidelines concerning PPIs and dementia to provide patients with decisions supported by evidence (Table **2**).

## 7 CHALLENGES IN DEMENTIA AND PPI RESEARCH

Existing evidence on dementia associated with PPIs is limited in a number of ways. Due to the prevalence of observational data in scientific research, establishing causality is difficult. Co-morbidities and polypharmacy, among other confounding variables, may have an impact on the observed associations. Prospective research is justified in employing rigorous methodology, extended follow-up periods, and an emphasis on potential confounding variables. Moreover, in order to investigate the underlying biological processes that contribute to PPI-associated dementia, mechanistic investigations are required [157].

## CONCLUSION

In conclusion, dementia stands as an urgent global health concern, affecting millions globally and imposing significant economic and caregiving burdens, particularly on women and in countries with low or middle incomes. This review highlights the emerging association between prolonged PPI usage and dementia, drawing attention to the potential link between cognitive decline and neurodegenerative conditions. Conflicting findings across different studies demonstrate the complex nature of this association. Long-term PPI use may be unsafe for our brains in various ways, such as changing the pH of brain cells as changing brain cells and causing Aβ peptides to build up. The treatment of elderly patients with PPI therapy should be personalized and cautious, involving alternative medications and gradual dosage reduction. Regular follow-up and comprehensive medication reviews are crucial for patient safety and holistic health, especially in the vulnerable geriatric population. Healthcare providers, particularly when caring for older adults, need to carefully evaluate the risks and benefits of PPI therapy, taking into account alternative treatments when appropriate.

## AUTHORS’ CONTRIBUTIONS

Zuber Khan (Z.K) wrote the original draft of the review, and Sidharth Mehan (S.M) and Ghanshyam Das Gupta (G.D.G) conceptualized and supervised the study., Mohd. Anas Saifi (M.A.S), Acharan S Narula (A.S.N), and Reni Kalfin (R.K) validated and helped in Review and Editing. All authors agree to be accountable for all aspects of work, ensuring integrity and accuracy.

## Figures and Tables

**Fig. (1):**
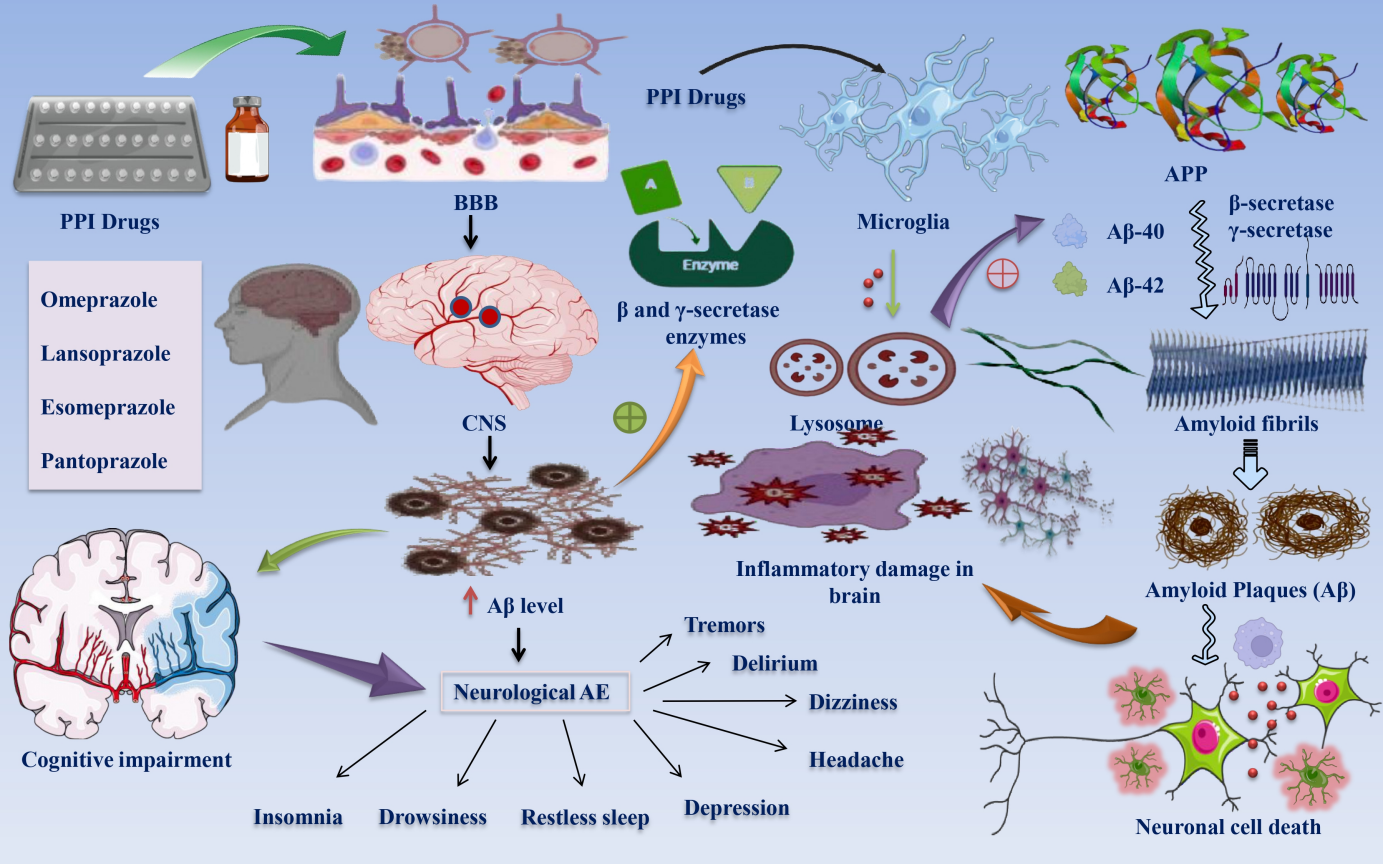
Effects of chronic PPI administration on CNS and Aβ plaque elevation and cognitive impairment. The diagram shows the capability of PPI medications to cross the BBB and enter the CNS compartment [6, 35]. Extended use of PPIs has noticeable impacts on cognitive processes while also causing neurological symptoms, including drowsiness, insomnia, tremors, migraines, and disturbed sleep patterns [38]. A greater quantity of Aβ plaques may potentially hinder the cognitive functioning of those affected [21]. PPI medications have been observed to increase Aβ levels through modulation of β and γ-secretase activity, thereby causing oxidative and inflammatory injury in the cerebral environment [22, 25]. In addition, PPI-associated Aβ enrichment may entail the regulation of Aβ degradation in the lysosomal compartments of microglial cells [31]. Enhanced levels of pathogenic Aβ40 and Aβ42 species are implicated in the amyloid fibril and plaque formation process. It is hypothesized that the increased abundance of Aβ constitutes a crucial factor in the death of neurons [26, 31]. **Abbreviations:** PPI: proton pump inhibitor; BBB: Blood-brain barrier; AE: Adverse effects; Aβ: Amyloid-β plaque.

**Fig. (2):**
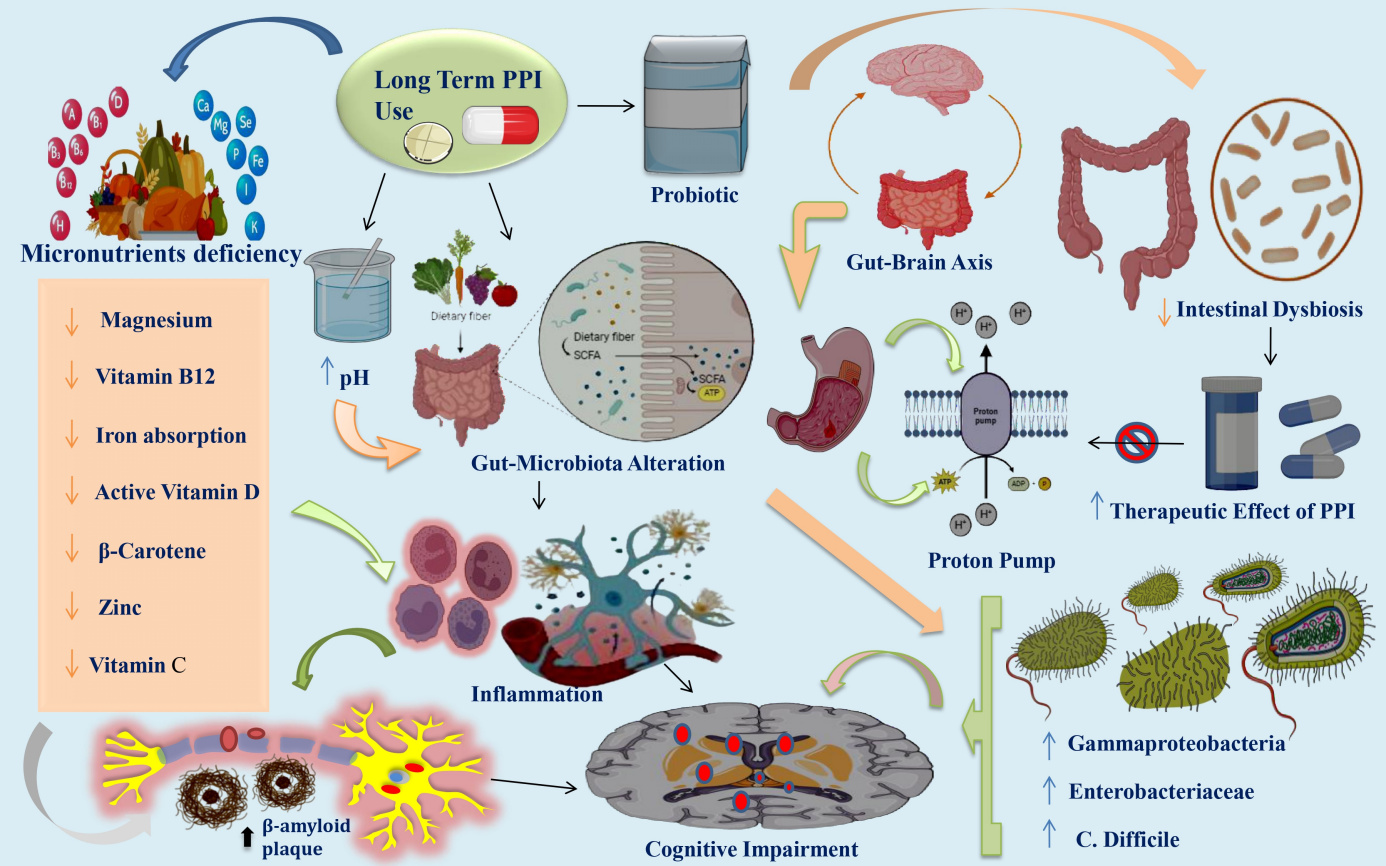
PPI-induced micronutrient deficiencies, gut-brain axis, and cognitive implications: an integrated perspective. The provided figure illustrates that extended use of PPIs is linked to adverse outcomes despite being widely recognized for their effectiveness and tolerability. Notably, these include reduced levels of essential micronutrients, such as magnesium, vitamin B12 deficiency, calcium and iron absorption, β-carotene, active vitamin D, zinc, and vitamin C [57-59]. A correlation exists between insufficiencies of these micronutrients and cognitive dysfunction and the development of Aβ plaques [55]. The gut-brain axis supports bidirectional communication between the enteric and central nervous systems by connecting the peripheral functions of the intestines with the affective and cognitive domains of the brain [39]. Extended use of PPIs, which suppress gastric acid, compromises the integrity of the intestinal barrier, leading to a modification in the composition of the microbiota in the small intestine and ultimately causing intestinal dysbiosis [51]. Prolonged administration of PPIs alters the composition of the gastrointestinal microbiome, distinguished by an increased abundance of bacterial taxa, including Gammaproteobacteria, Enterobacteriacea, and Clostridium difficile [59]. The efficacy of PPI medications is enhanced through the usage of probiotic nutrition. In addition, probiotic supplements have shown promise in alleviating intestinal dysbiosis and the negative consequences of extended use of PPI [52]. The activation of microglia, which are innate immune cells of the brain, by Aβ plaque deposits induces an inflammatory response and initiates cognitive impairments in the central nervous system [68]. **Abbreviations:** PPI: Proton pump inhibitor; Aβ: Amyloid-β plaque.

**Table 1:** Clinical studies characteristics of long-term PPI use and dementia risk findings.

**S. No.**	**Study Title**	**Study Design**	**Sample Size (PPI/Non-PPI)**	**Duration of PPI**	**Key Findings**
1.	Bonomini *et al.*, 2015 [100]	Prospective cohort	13,864 patients	9-14 years follow up	Unable to find convincing evidence of PPI-associated dementia risks in the study
2.	Miller, 2018 [123]	Retrospective cohort	36,360 (PPI) 99,362 (Non-PPI)	13 years	Observations indicate a slight increase in the risk of non-AD dementia.
3.	Moledina and Perazella, 2016 [21]	Prospective cohort	2950 (PPI) 70,729 (Non-PPI)	5.4 years	Found statistical link between the use of PPI and risk of dementia
4.	Torres -Bondia *et al.*, 2020 [124]	Retrospective cohort	7342 (PPI)	3 years	An increased risk of PPI-associated dementia was found
5.	Park *et al.*, 2018 [125]	Retrospective cohort	6584 (PPI)	5 years	PPI users found at higher risk of dementia
6.	Chen *et al.*, 2020 [126]	Retrospective cohort	11828 (PPI) 11828 (Non-PPI)	10 years	A significant association between PPI users and dementia was found
7.	Zhang *et al.*, 2022 [12]	Retrospective cohort	183,968 (PPI) 131110 (Non-PPI)	10.9 years	Unable to find the link between the use of PPI and increased dementia risk
8.	O’Brien and Wong, 2011 [27]	Prospective study	402 (PPI) 3082 (Non-PPI)	7.5 years	PPI was not found to be linked with the risk of dementia
9.	Welu *et al.*, 2019 [127]	Retrospective cohort	10,533 (PPI)	5 years	PPI was not significantly found to increase the dementia risk
10.	Huang *et al.*, 2019 [128]	Multicenter double-blind, randomized controlled trial	8791 (PPI) 8807 (Placebo)	3 years	No association between PPI and dementia was found
11.	Moayyedi *et al.* 2019 [129]	Prospective cohort	**LSADT cohort** 299 (PPI) 3316 (Non-PPI) **MADT cohort** 262 (PPI) 4001 (Non-PPI)	2-10 years follow up	No link was found between chronic PPI use and cognitive score or decline.
12.	Cooksey *et al.*, 2020 [13]	Prospective cohort	Cases: 2505, including 932 (AD), 524 vascular dementia (VaD)	9 years	Chronic use of PPI was found to be associated with incident dementia
13.	Varghese *et al.*, 2022 [11]	Meta-analysis	6 cohort study	0.67-9 years follow up	PPI was not significantly associated with dementia
14.	Wod *et al.*, 2018 [130]	Observational longitudinal study	884 (Always PPI user) 1925 (Intermittent PPI user) 7677 (Non-PPI user)	10 years	PPI use was linked with lower cognitive function decline risk
15.	Goldstein *et al.*, 2017 [131]	Meta-analysis of 11 observational studies	158,954 (PPI users) 483,995 (Non-PPI)	5-10 years	No evidence has been discovered to be linked between PPI and increased dementia risk.

**Note:** The given table collects data from a variety of clinical studies looking into the link between long-term PPI use and dementia. The research uses a variety of designs, including multicenter double-blind, randomized controlled trials, prospective and retrospective cohorts, and meta-analyses. Participants in the studies used PPIs for durations ranging from about 2 to 14 years, and the sample sizes were widely dispersed. The primary findings are inconsistent; although certain research indicates a greater chance of dementia associated with PPI use, other studies find no conclusive evidence of a significant correlation, if any, between PPI use and dementia risk. These conflicting findings highlight the need for additional investigation into the possible causes of the association between PPI usage and dementia. **Abbreviations:** PPI: proton-pump inhibitor, AD: Alzheimer’s disease, VaD: vascular dementia, LSADT: Longitudinal Study of Aging Danish Twins, MADT: Middle-Aged Danish Twin study.

**Table 2:** Treatment drugs used for the various forms of dementia.

**S. No.**	**Drug Name**	**Mechanism of Action**	**Adverse Effects**	**Dose and Routes**	**Types of Dementia**	**References**
1.	Donepezil	AChEIs (Acetylcholine esterase inhibitors)	Nausea, vomiting, diarrhea, headache	5-10 mg once daily, orally	Mixed dementia	[133, 134]
2.	Rivastigmine	AChEIs	Nausea, vomiting, diarrhea, appetite loss, diarrohea, insomnia, muscle cramps	6-12 mg daily, orally or 9.5 mg transdermally	Mild to moderate dementia	[135, 136]
3.	Galantamine	AChEIs	Nausea, vomiting, diarrhea, weight loss, Abdominal pain, dizziness, confusion, agitation, insomnia, headache, UTI, and rarely severe bradycardia	16-24 mg once daily, orally	Mild to moderate dementia	[137, 138]
4.	Memantine	-NMDA Antagonist -↓Cognitive decline	Dizziness, Nausea, headache, constipation, Insomnia, Agitation	20 mg daily, orally	Dementia with Lewy bodies	[139-141]
5.	Acetyl-L-Carnitine	↑Ach synthesis ↑ protein & membrane phospholipids	vomiting, diarrhea, increased appetite, agitation, insomnia	500-1500 mg twice daily Orally, IV or I.M	Mild to moderate dementia	[142-144]
6.	Huperzine A	AChEIs	Dry mouth, mild bellyache, diarrhea, sweating, mild nausea, dizziness	0.15 mg daily, orally	Vascular dementia	[145, 146]
7.	Quetiapine	-Anti-psychotic -Rebalance dopamine and serotonin in the brain	Sedation, dizziness, weight loss, Lethargy, headache	100-200 mg, usually once daily	Behavioral and psychological symptoms of dementia	[147, 148]
8.	Aducanumab	Anti-amyloid antibody intravenous	Edema, headache, diarrhea, confusion. falls	≤30 mg/kg I.V. infusion,	Mild dementia or (MCI) due to AD	[149, 150]
9.	Lecanemab	anti-amyloid antibody intravenous	infusion-related reactions, cerebral microhemorrhage, cerebral macrohemorrhage, or superficial siderosis, headache, and falls	10mg/kg biweekly, I.V. infusion therapy	MCI or mild dementia due to AD	[151, 152]
10.	Brexpiprazole	-Third-generation antipsychotic -Potent binding to 5-HT2A, 5-HT1A and alpha1B receptors	Headache, insomnia, UTI, dizziness	1-2mg/day orally	Agitation associated with dementia due to AD	[153, 154]
11.	Suvorexant	Orexin receptor antagonist	Somnolence, headache, fall, dry mouth, diarrohea, sleep paralysis, agitation, confusion	10-20mg, orally	Mild to Moderate dementia	[155, 156]

**Note:** The table and summarizes the various drugs used to treat different types of dementia. Essential details such as the type of drug, modes of action, side effects, dose and administration methods, and the specific types of dementia that the medications are used for are included. Acetylcholine esterase inhibitors, such as galantamine, rivastigmine, and donepezil, reduce the symptoms of mild to moderate dementia. Memantine is used for Lewy body dementia, but it operates through a distinct mechanism. Furthermore, the table presents novel treatments for moderate cognitive impairment or mild dementia caused by AD, including Aducanumab and Lecanemab, both of which are anti-amyloid antibodies. Based on the type and severity of dementia, the given information may help medical practitioners identify the appropriate medications, enabling them to make well-informed and personalized treatment decisions. **Abbreviations:** AChEI: Acetylcholinesterase inhibitors, NMDA: N-Methyl-D-Aspartate, MCI: Mild cognitive impairment, UTI: Urinary tract infection, Ach: Acetylcholine.

## LIST OF ABBREVIATIONS

PPI: Proton Pump Inhibitor
GERD: Gastroesophageal Reflux Disease
Aβ: Amyloid Beta
AD: Alzheimer’s Disease
APP: Amyloid Precursor Protein
AGS: American Geriatric Society
BBB: Blood-Brain Barrier
CNS: Central Nervous System
AUC: Area Under the Curve
BACE: Beta-site APP Cleaving Enzyme
SIBO: Small Intestinal Bacterial Overgrowth
DDD: Defined Daily Dose
AChEI: Acetylcholinestrase Inhibitor
MCI: Mild Cognitive Impairment
UTI: Urinary Tract Infection
MCI: Mild Cognitive Impairment
FTD: Frontotemporal Dementia
FTLD: Fronto-temporal Lobar Degeneration
